# Hoosier Sport Re-Social: a protocol for developing a biopsychosocial body satisfaction intervention in rural Indiana

**DOI:** 10.1186/s40814-025-01695-5

**Published:** 2025-08-25

**Authors:** Janette M. Watkins, Janelle M. Goss, Vanessa M. Martinez Kercher, Cassandra J. Coble, Nicole E. Werner, R. Glenn Weaver, Kyle A. Kercher

**Affiliations:** 1 Department of Kinesiology, School of Public Health-Bloomington, Indiana University, Bloomington, IN USA; 2 Program in Neuroscience, College of Arts and Sciences, Indiana University, Bloomington, IN USA; 3 Logan University, Chesterfield, MO USA; 4 Department of Health & Wellness Design, School of Public Health-Bloomington, Indiana University, Bloomington, IN USA; 5 Department of Exercise Science, Arnold School of Public Health, University of South Carolina, Columbia, SC USA

**Keywords:** Body image, Body satisfaction, Early-intervention

## Abstract

**Background:**

Body dissatisfaction is a growing concern among adolescent females, particularly those living in rural communities where access to supportive mental and physical health resources is limited. High levels of social media use and low engagement in structured physical activity are linked to negative body image, elevated anxiety, and reduced overall well-being in this population. The Hoosier Sport Re-Social intervention was developed to address these challenges by integrating sport participation, social media literacy, and mental skills training in a community-based program designed for adolescent girls.

**Methods:**

This study follows the Standard Protocol Items: Recommendations for Interventional Trials (SPIRIT) guidelines and is structured around three aims. First, we will conduct a cross-sectional study with adolescent girls in grades 6 through 9 to examine the relationship between body satisfaction, physical activity levels, and social media use. Second, we will develop the Hoosier Sport Re-Social intervention using a co-design process that actively involves adolescents, parents, and school staff to ensure relevance and feasibility. Finally, we will conduct a mixed-methods pilot study in two rural middle and high schools to assess feasibility and acceptability. The intervention will be implemented within physical education and health classes over a 6-week period. Primary outcomes will include feasibility indicators such as recruitment, retention, fidelity, and participant engagement. Secondary outcomes will include measures of body satisfaction and social media literacy, while exploratory outcomes will examine changes in psychosocial factors and physical literacy.

**Discussion:**

This study will provide important insights into the acceptability and practicality of delivering a biopsychosocial, school-based intervention targeting body dissatisfaction among rural adolescent girls. Findings will inform future efforts to scale the program and evaluate its effectiveness in improving mental, physical, and cognitive health outcomes.

**Trial registration:**

This trial was prospectively registered with ClinicalTrials.gov (NCT06556719).

**Supplementary Information:**

The online version contains supplementary material available at 10.1186/s40814-025-01695-5.

## Background

Fifty-three percent of American girls are “unhappy with their bodies,” with 46% of 9–11-year-olds actively dieting and reporting body dissatisfaction (generally defined as a negative subjective evaluation of one’s body size, shape, or overall appearance) [1]. This body dissatisfaction is correlated with the development of life-threatening mental health issues (e.g., body dysmorphia, eating disorders, self-harm) [2, 3]. Body dissatisfaction in adolescence is associated with poorer quality of life [4] due to factors such as negative affect [5] and self-talk [6], as well as low self-esteem [7, 8] and self-acceptance [6].

Social media use during adolescence creates potentially serious complications for mental health. The use of social media during adolescence is linked to a significant 189% rise in U.S. hospital admissions for non-fatal self-harm among adolescent females, which has been correlated to increases in body dissatisfaction [9, 10]. Furthermore, there has been a more than 151% [11] increase in suicide rates among adolescent females, and this is associated with the rising trend of increased social media use [12].

Social media significantly impacts adolescent female body image, creating unrealistic appearances through edited images and messages [13–18]. Key issues include exposure to distorted self-images, prolonged exposure to edited content, and unrealistic body portrayals [17, 19, 20]. Importantly, the daily average of screen time for adolescents is 6 to 9 h, and more screen time is correlated with greater body dissatisfaction [21, 22]. Furthermore, age significantly influences the relationship between social media use and body satisfaction, as highlighted in a recent systematic review [23], with younger females being at greater risk of body dissatisfaction due to social media.

Reducing exposure to social media in adolescent females should be a public health priority. Indeed, research underscores the vulnerability of adolescent females, emphasizing the need for targeted interventions and support for body image and social media usage [20], such as supportive school environments teaching media literacy [13, 24]. Currently, research supports multifaceted classroom interventions spanning at least 1 to 4 weeks to address body satisfaction and social media literacy [25]. Despite the promising potential of this work, interventions to improve social media literacy show only a limited effect, and few interventions emphasize targeting populations with increased health disparities (e.g., rural populations) [25, 26]. A recent systematic review on physical activity and healthy digital media usage revealed a significant research gap, with the majority of studies conducted only in high-income areas, with limited work in rural populations [27]. There remains a need for research in low- and middle-income populations, as there is limited available data on the necessity for comprehensive strategies to achieve behavioral change in school-aged children and adolescents [28, 29].

Adolescence, a crucial developmental stage for body image, requires interventions educating at-risk females about social media literacy and promoting positive body image (a holistic appreciation of one’s body, encompassing respect for its functionality, acceptance of its appearance, and the rejection of narrow beauty ideals) [19, 30]. Structured physical activity participation can be a powerful tool to improve body satisfaction in adolescent females [31–33]. In the present study, structured physical activity is defined as any activities that are planned in a formal setting to improve physical fitness or sport-related skill development (e.g., sports, workouts, fitness classes) [34]. Research demonstrates that sports can counteract body dissatisfaction in adolescent females [35]. Similarly, female adolescents who do not participate in sports/physical activity tend to have significantly lower levels of body satisfaction when compared to those who are actively involved in sports [33] or structured physical activity [31–33]. The link between structured physical activity and increased body satisfaction may be influenced by an elevated sense of body appreciation following exercise [31–33]. Additionally, improved physical literacy is associated with increased physical activity and decreased screen behavior [36, 37], which may help reduce social media usage.

**Fig. 1:**
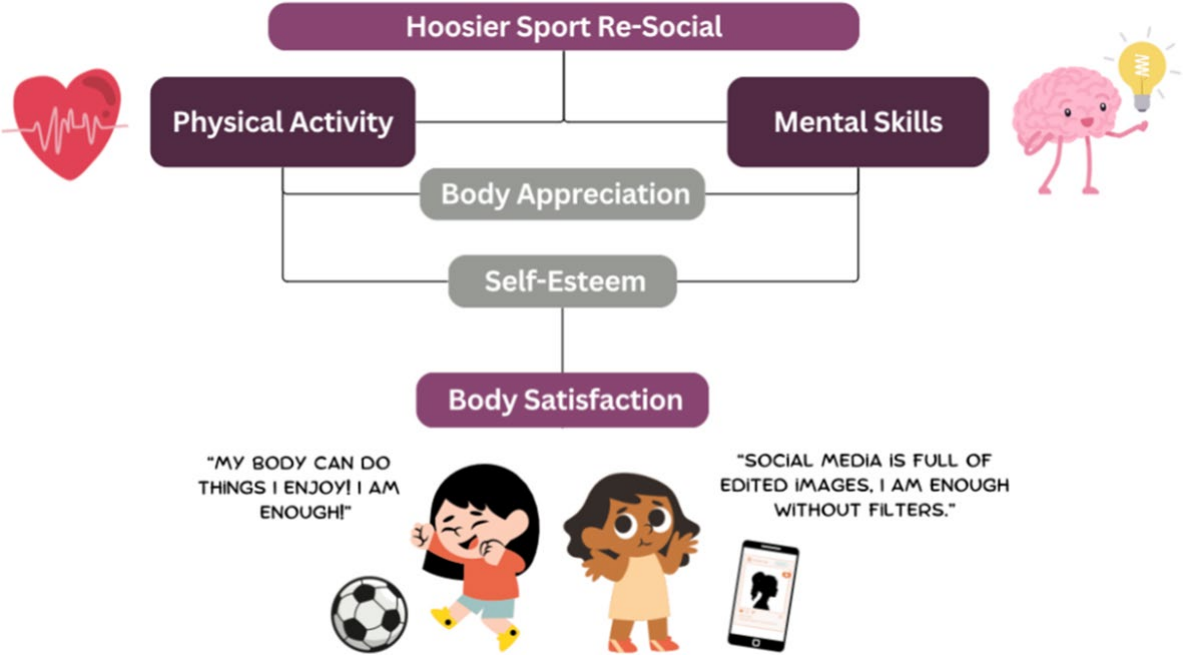
Hoosier Sport Re-Social goal

Despite the benefits of structured physical activity, alone it may not be sufficient to completely counteract the harmful effects of continuous exposure to negative body image messages prevalent on social media [33]. However, the addition of mental skills training may enhance the intervention’s potential. Mental skills training (e.g., goal setting, positive self-talk) is a helpful, age-appropriate, and easily accessible method for shifting the perspective of adolescent girls toward improved body satisfaction [38]. Engaging in just one session of positive self-talk has been shown to reduce body dissatisfaction and increase the intention to practice body positivity [38, 39]. Additionally, self-affirmations are a proven technique to mitigate the negative effects of body-image concerns induced by social media [40]. Hence, mental skills training can be a valuable addition to a sports-based program targeting body satisfaction in adolescent girls. Figure 1 illustrates this model. The addition of mental skills training may enhance the intervention’s potential effectiveness. Mental skills training (e.g., goal setting, positive self-talk) is a helpful, age-appropriate, and easily accessible method for shifting the perspective of adolescent girls toward improved body satisfaction [38]. Engaging in just one session of positive self-talk has been shown to reduce body dissatisfaction and increase the intention to practice positive body image [38, 39]. Additionally, self-affirmations are a proven technique to mitigate the negative effects of body-image concerns induced by social media [40]. Hence, mental skills training can be a valuable addition to a sports-based program targeting body satisfaction in adolescent girls. Figure 1 illustrates this model.

The primary objective of the proposed study is to develop and evaluate the feasibility of a biopsychosocial intervention designed to improve body satisfaction among adolescent females. This intervention, Hoosier Sport Re-Social,^1^ integrates mental skills training and social media literacy within a sport-based youth development program tailored to the unique needs of adolescent girls in rural schools in the USA. Our central hypothesis is that the intervention, developed through a collaborative co-design process with adolescents, parents, and teachers, will demonstrate both feasibility and acceptability in the rural middle school context. Specifically, we anticipate that the intervention will be practical to implement and well received by the target population, addressing key factors influencing body satisfaction and supporting positive outcomes in physical activity and social media engagement. By focusing on these areas, the study aims to provide a scalable model for improving body satisfaction and promoting healthier behaviors among adolescent females in similar rural settings.

## Methods/design

### Overview

This study is structured into three distinct phases to address body image perceptions and develop interventions for adolescent females enrolled in under-resourced rural middle and high schools (grades 6–9). The first phase, AIM 1, involves conducting a comprehensive needs assessment through community-engaged research to build foundational knowledge about body image, social media usage, and physical activity levels among rural adolescents. This phase aims to identify current physical activity levels and assess facilitators and barriers to positive body image within the adolescents’ context.

In the second phase, AIM 2, the focus shifts to co-designing the Hoosier Sport Re-Social intervention with key community stakeholders. This involves collaborating with adolescents, parents, and teachers to develop a prototype multi-level intervention and implementation protocol. Participant involvement is central to the Hoosier Sport Re-Social protocol. Adolescents, parents, and teachers are not only research participants but also co-creators of the intervention. Through a series of structured co-design sessions, these stakeholders actively contribute to identifying problems, generating solutions, and refining the final prototype, ensuring the program is contextually relevant and responsive to community needs. The goal is to create an intervention that is responsive to the needs and preferences of adolescents, specifically targeting the promotion of a positive body image.

**Fig. 2:**
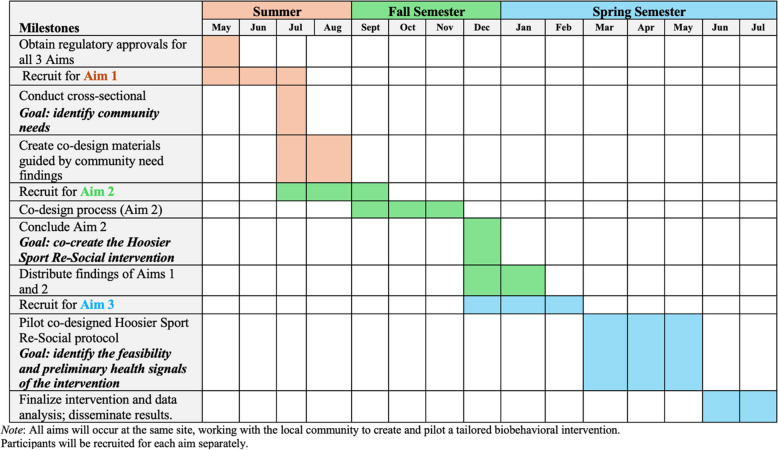
Proposed study timeline

The third phase, AIM 3, involves piloting the Hoosier Sport Re-Social intervention with a sample of 50 adolescents, divided into 25 in the intervention group and 25 in the control group. This phase will evaluate the feasibility and acceptability of the intervention, assessing both trial-related and intervention-specific feasibility indicators. Feasibility refers to the extent to which an intervention can be successfully delivered in a specific setting, considering factors such as recruitment, retention, acceptability, adherence, and implementation fidelity. The overarching hypothesis of the study is that the successful completion of these three phases will result in a feasible and effective intervention protocol for promoting a positive body image among adolescent females in rural settings. Figure 2 provides a timeline of these aims.

## Conceptual framework

**Fig. 3:**
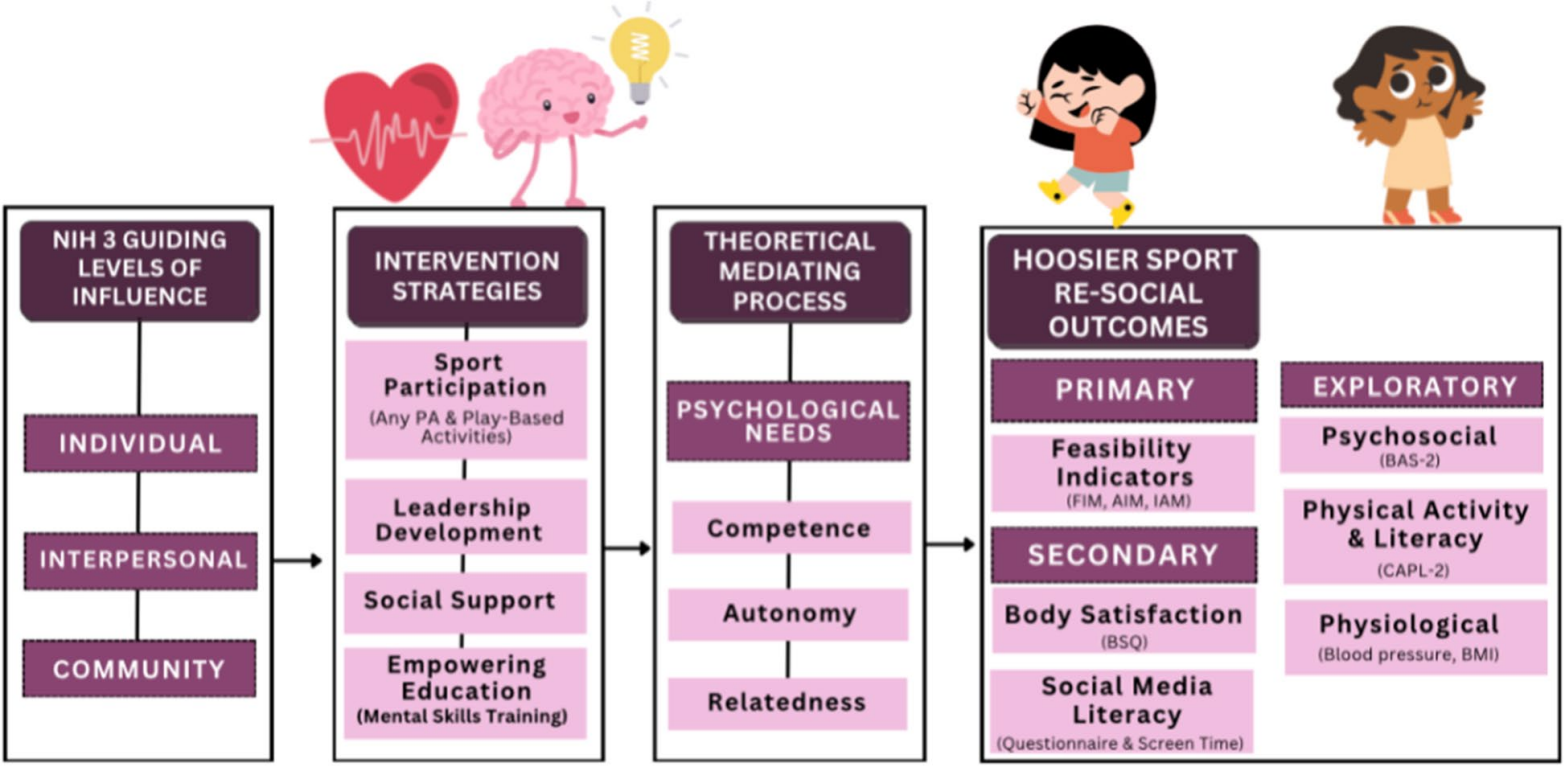
Conceptual framework

The study’s foundation rests on three key theoretical elements: the Basic Psychological Needs mini-theory within self-determination theory (SDT) [41], the biopsychosocial model [42], and Obesity-Related Behavioral Intervention Trials (ORBIT) model [43]. SDT asserts that autonomy, competence, and relatedness are essential for human motivation and well-being; the biopsychosocial model suggests that health and illness are shaped by biological, psychological, and social factors, underscoring the interconnected nature of these dimensions in comprehending human health and disease; meanwhile, ORBIT furnishes a structured approach for crafting and assessing behavioral interventions aimed at obesity, merging insights from health psychology, behavioral economics, and implementation science to optimize intervention efficacy and longevity. Each of these models guided the methodology development for the study’s three aims. Specifically, the Basic Psychological Needs mini-theory within SDT aids in predicting and examining outcome-influencing factors. The biopsychosocial model assists in describing and interpreting findings without directly predicting outcomes; and the study is guided by the systematic approach to intervention development and testing posited by the ORBIT model. Figure 3 provides an overview of the conceptual framework.

### Aim 1 (needs assessment): assess adolescent females’ views on social media, physical activity, and factors impacting positive body image in the community

#### Aim 1: design

The study’s initial phase involves conducting a community-engaged research needs assessment (ORBIT Phase Ia) to identify the needs, goals, opportunities, and assets related to physical activity within the community. This phase aims to build on existing knowledge about the population-level effects of social media on psychosocial factors by gaining a detailed understanding of how these factors impact needs in rural populations. To achieve this, we will perform a comprehensive needs assessment focused on body image by surveying adolescents, parents, teachers, and school administrators. The survey will utilize a multilevel design that addresses individual, interpersonal, and community-level influences. The insights gained from this needs assessment will provide a foundational understanding of the interactions between social media, physical activity, and body satisfaction within the current school setting. Additionally, this assessment will inform future evaluations across additional school partners, enhancing the overall understanding of these dynamics in rural contexts.

AIM 1 will be conducted in one rural public school district in Indiana, including both the middle school and high school campuses. These schools serve students in grades 6 through 12 and are located in an under-resourced, predominantly White community where all students qualify for free and reduced-price meals. While we will not stratify recruitment by grade, we will aim to include students from each eligible grade level (6th–9th) to capture a range of developmental perspectives. Grade-level representation will be monitored during enrollment, and descriptive analyses will examine potential differences by grade to inform future iterations of the program. Recruitment is conducted in collaboration with school staff and includes multiple strategies to reach eligible students and parents. For students, in-school announcements and classroom visits by research staff are used to explain the study and distribute take-home flyers. Parents are informed through printed flyers sent home with students and direct communication facilitated by teachers or school administrators.

#### Aim 1: setting and sample

We have partnered with a rural Midwestern middle school and high school, serving predominantly White students from under-resourced communities. Given the district's high poverty rates, the entire student body qualifies for free and reduced meals. Our data collection plan involves surveying a sample of 50 rural students. This sample size has been selected to ensure feasibility while providing a sufficiently large cohort for outcomes to approximate normal distributions, in accordance with the central limit theorem [44, 45]. Our inclusion criteria for participants are as follows: students must be enrolled in middle or high school, specifically entering grades 6 through 9 in the spring 2024 semester. Additionally, participants must have parental consent and provide their own assent to participate in the survey. For AIM 1, the focus will be on exploring body image issues among female students within the rural youth sample. However, males will be included to allow for comparison between biological sexes, and to further guide intervention development for greater inclusion.

#### Aim 1: procedure

##### Youth survey

After securing parental consent, we will obtain assent from adolescents to ensure they fully understand the study’s purpose, requirements, and potential risks or benefits. Parental consent will be gathered remotely via an informed consent document distributed through Qualtrics survey software. Adolescent assent, along with survey administration, will be conducted in-person using Qualtrics. This approach is designed to enhance compliance and ensure that adolescents have a clear understanding of the study. The survey measures will include demographics, the Canadian Assessment of Physical Literacy (CAPL-2) [46], Basic Psychological Need Satisfaction and Frustration Scale (BPNSFS) [47, 48], Body Appreciation Scale (BAS-2) [49], and Body Shape Questionnaire (BSQ) [50, 51]. Additionally, youth-tailored questions related to social media use will be used. The survey also includes an item assessing parental control of social media, asking participants whether their parent or guardian monitors or limits their account access or screen time. This item will support exploratory analyses of how parental involvement relates to digital behavior and psychosocial outcomes. The survey is anticipated to take 15 min. See the *Measures* section for additional details and see Supplementary material 1 for the complete youth survey.

#### Aim 1: measures

##### Physical activity

The CAPL-2 will be used to assess self-reported physical activity behaviors and physical literacy in adolescents. The CAPL-2 assesses physical activity during physical education class, recess, lunch, right after school, evening, weekends, and spare time. The CAPL-2 is 22-items [52, 53]. Additionally, the CAPL-2 has been shown to be reliable for measuring physical literacy in adolescents [52]. Additionally, accelerometers (Axivity, AX3) will be used to objectively measure PA via daily steps. The AX3 has shown high validity in adolescent populations for measuring PA [54].

##### Psychological needs

Adolescents will rate the satisfaction of their psychological needs (i.e., autonomy, competence, relatedness) with the Basic Psychological Need Satisfaction and Frustration Scale (BPNSF). The BPNSFS consists of 24 items with scores on a 5-point Likert scale ranging from “strongly disagree” to “strongly agree.” Twelve items assessed satisfaction (with four questions each for autonomy, competence, and relatedness), and twelve items assessed frustration (with four questions each for autonomy, competence, and relatedness). The BPNSFS has demonstrated adequate internal consistency with Cronbach’s alpha coefficients of global basic psychological need satisfaction of 0.9, 0.80 for autonomy satisfaction, 0.81 for competence satisfaction, and 0.81 **for** relatedness satisfaction [47, 48].

##### Perceptions of body

The BSQ will be used to assess body satisfaction, comparison to others, and emotional aspects surrounding body weight. The BSQ has a Cronbach’s alpha value of.93 in female populations [55]. Additionally, the Body-Appreciation Scale (version 2) (BAS-2) will be used to assess body appreciation levels. The BAS-2 assesses body appreciation across a series of levels: *body competence, body acceptance, body respect,* and *body protection*. The BAS-2 has consistently exhibited robust internal consistency. A strong internal consistency is reflected in Cronbach’s alpha values surpassing 0.80 [49], a benchmark commonly recognized as indicative of the scale’s reliability.

##### Social media usage

Currently, there exists a lack of validated tools for quantifying social media usage. To achieve an objective assessment of social media usage, this study evaluated the daily average screen time and usage of social media applications. Data will be extracted directly from participants’ smartphones, utilizing their settings and screen time features. Participants will be asked to enter this data themselves to protect their personal information. Additionally, the Bergen Social Media Addiction Scale (BSMAS) [56] will be used to objectively measure levels of addiction to social media. The BSMAS is an 8-item questionnaire, derived from the Facebook Intrusion Questionnaire (FIQ), which measures Facebook addiction through behavioral addiction symptoms (e.g., withdrawal, relapse, reinstatement, and euphoria). However, the BSMAS assesses multiple social media platforms. The BSMAS has demonstrated reliable psychometric properties in adolescent females (α: 0.86) [57].

#### Aim 1: sample size

To determine the appropriate sample size for our study, we conducted power analyses using G*Power. For the initial linear regression model, a priori testing was conducted with a specified moderate effect size of 0.6, three predictors, and a significance level of 0.05. The analysis indicated that a sample size of 29 participants would be required to achieve adequate statistical power. A moderate effect size was deliberately selected to address a common limitation in feasibility studies, wherein inadequate sample sizes, low statistical power, and the potential for generating misleading results are prevalent [58]. A decision was made to avoid selecting a large effect size in order to maintain alignment with the intended scope of a feasibility study and to steer clear of encroaching upon the objectives more commonly associated with a randomized clinical trial [59]. Likewise, applying the same parameters to the logistic regression model, we also determined that a sample size of 29 participants would be necessary. Consequently, our research team aims to recruit a total of 30 or more participants for the implementation of AIM 1, ensuring that our study is appropriately powered to detect meaningful effects, and accounting for potential dropouts.

#### Aim 1: data analysis

For descriptive statistics, we will compute frequencies and percentages for each categorical variable and calculate means and standard deviations for continuous variables. Quantitative analyses will be performed in R 4.0.3 [60]. In collaboration with the Indiana University Center for Survey Research, the research team will review qualitative and quantitative responses to identify general patterns and main themes. The results of the survey analysis will be reviewed by the research team and considered for incorporation into future school surveys. Results from Aim 1 will be used to inform Aim 2 co-design session topics and to guide co-design session agendas.

Qualitative data from the co-design sessions will be analyzed through thematic analysis to identify unique community needs. To conduct this analysis, the team of researchers will complete six phases (familiarization with data, generation of codes, searching for themes, reviewing themes, defining and naming themes, and producing the report) as outlined by Braun and Clarke (2006). Quantitative data will be assessed using linear regression models to explore the relationships between key variables, with emphasis on physical activity levels, social media usage, and body satisfaction. For the linear regression models, model 1 will explore Body Satisfaction Prediction. The dependent variable will be body satisfaction (measured by the BSQ), and the independent variables will be body appreciation (BAS-2 scores) and body-guided behavior (MAIA-Y scores). The primary regressions will be: (Table 1).

**Table 1:** Proposed regressions for analysis

Outcome	Independent Variables	Purpose
BSQ (Body Image Score)	MET, Sex	To assess the relationship between physical activity (MET) and sex on body image.
BSQ	BSMAS, Screen Time, Sex	To determine the impact of problematic social media use (BSMAS), screen time, and sex on body image.
BAS-2 (Body Appreciation)	MET, Sex	To explore the effect of physical activity and sex on body appreciation.
BAS-2	BSMAS, Screen Time, Sex	To analyze how social media use and screen time relate to body appreciation, accounting for sex.
BSQ	BAS-2, Sex	To evaluate the association between body appreciation and body image, adjusting for sex.
GAD (Anxiety)	Screen Time, BSMAS, Sex	To assess how screen time and social media use relate to anxiety, accounting for sex.
GAD	MET, Sex	To investigate the relationship between physical activity and anxiety, adjusting for sex.
MET	CAPL-2, Sex	To examine the effect of physical literacy (CAPL-2) and sex on physical activity levels (MET).
BSQ	CAPL-2, Sex	To determine the relationship between physical literacy and body image, adjusting for sex.
BAS-2	CAPL-2, Sex	To explore how physical literacy influences body appreciation, accounting for sex.

All models will use linear regression. For each model, we will report unstandardized beta coefficients, standard errors, 95% confidence intervals, and p-values. Variables will be checked for assumptions of linearity, normality, and multicollinearity prior to analysis

### Aim 2: Co-create the Hoosier Sport Re-Social intervention in collaboration with White River Valley middle school children and adults

#### Aim 2: design

The study’s second aim is to co-design a prototype intervention and implementation protocol for Hoosier Sport re-social (ORBIT Phase Ia). Aim 2 will yield a protocol for both an intervention and implementation strategy. From here forward, “intervention protocol” will be used to describe the inclusive document detailing intervention components and implementation components (see Fig. 2 for more details). This phase aims to gain greater comprehension of the body satisfaction-based needs of youth from primarily low-socioeconomic rural backgrounds by focusing on individual, interpersonal, and school-level influences. The process will follow an established 5-step participatory co-design protocol [61, 62], consisting of sessions dedicated to problem identification, solution generation, solution evaluation, operationalization, and prototype evaluation. This participatory approach aims to empower both children and adults (e.g., parents [*n* = 3] teachers [*n* = 2]) to contribute to designing the *Hoosier Sport Re-Social* body-positivity intervention protocol. We plan to recruit two separate co-design teams, each comprised of *n* = 5 adults and *n* = 5 adolescents. The completion of these participatory co-design sessions will culminate in a testable prototype intervention and implementation protocol to be tested in Aim 3.

#### Aim 2: setting and sample

We plan to form two co-designer groups, one comprised of parents and the other children, with each group consisting of *n* = 5 recommended group size for participatory design [63]. This odd number of participants allows for a majority vote to resolve ties between design alternatives within the group. Recruitment of adults and children will occur through parent/guardian meetings and school administrators’ weekly newsletters. While utilizing convenience sampling due to limited sample size and time constraints, efforts will be made to enroll an approximately equal distribution of 50% female and 50% male participants, including those regularly involved in social media and those who are not. To be eligible for participation, children must be enrolled in middle or high school, entering 6th to 9th grade, and have parental consent while committing to attend all 5 co-design sessions. Adults eligible for inclusion must be a parent/guardian of a current 6th through 9th-grade student at the school and be willing to attend all 5 co-design sessions.

#### Aim 2: procedure

**Fig. 4:**
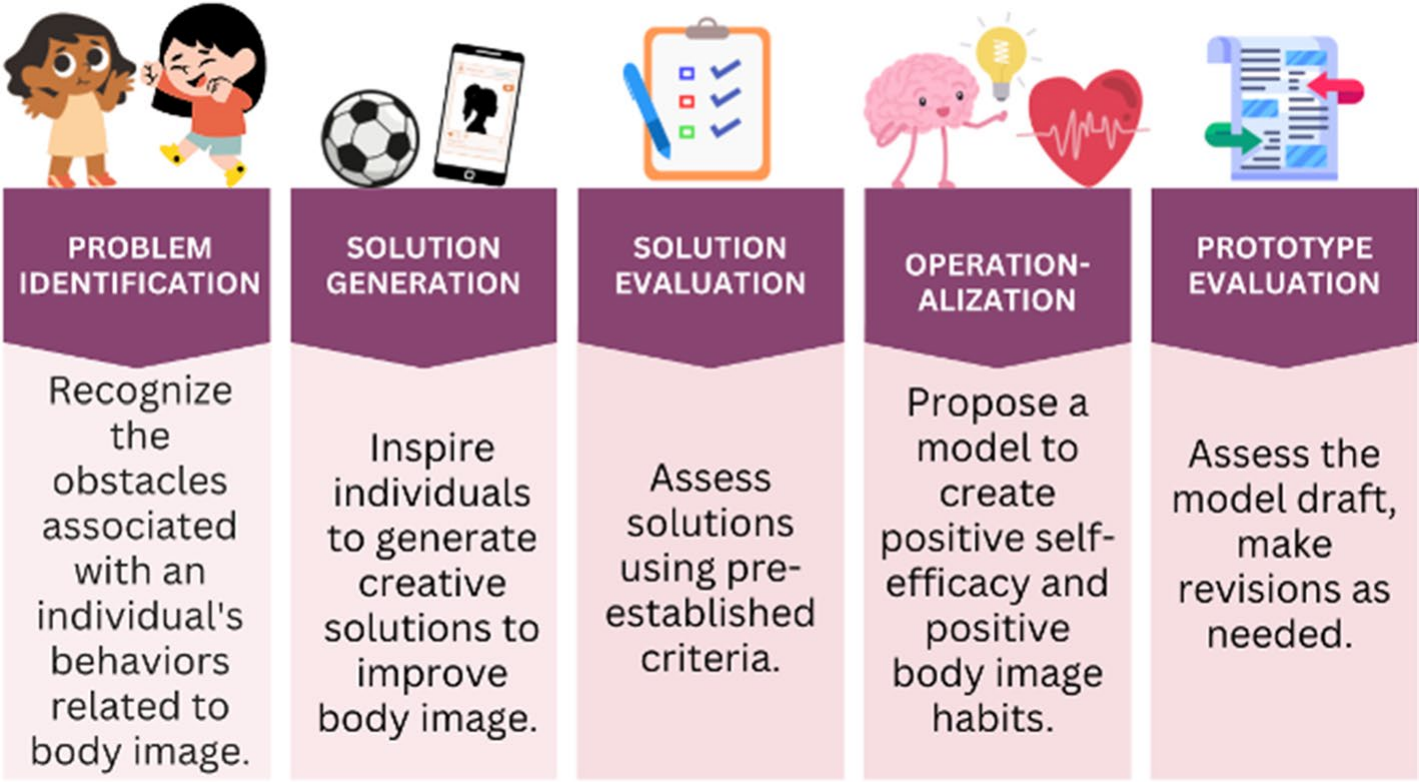
Co-design session goals

The two design teams will engage in a series of five co-design sessions spread across 3 months, with approximately 2 weeks between sessions. The adolescent group will initiate the process, while in parallel, the adult group will alternate sessions with the adolescent group (e.g., adolescent session, adult session, and so forth). The adult group will collaborate with the study team to review and refine the prototype developed by the children, aiming to retain as many adolescent-derived components as possible. This simultaneous yet alternating co-design approach empowers adolescents to incorporate essential concepts such as fun and enjoyment, while allowing adults to refine the intervention protocol for feasibility and practicality. Please see Fig. 4 for additional details.

Facilitated by an experienced team member trained in group coaching and discussions, each session will be guided by open-ended questions aligned with specific session goals. For instance, the first session will focus on understanding challenges related to adolescent body image behaviors. The iterative design process involves collectively understanding challenges, generating various solution ideas, and progressively developing a detailed intervention protocol. The facilitator will encourage discussion, interpretation, and respectful debate among team members while ensuring progress. Insights from Aim 1’s body image-related needs, goals, opportunities, and assets will be integrated into discussions by sharing survey results with co-designers.

The research team will collect observation notes and audio recordings to analyze the design teams’ collaborative efforts. These records will capture participants’ conversations, thought processes, and collaborative endeavors in generating and grouping intervention design solutions. Facilitators will guide participants in generating and collaborating on intervention protocol design solutions. Each session will last 60–75 min, and observation notes will be analyzed alongside audio recordings. In the final session, teams will evaluate the feasibility, acceptability, and appropriateness of the prototype Hoosier Sport Re-Social protocol using adapted measures tailored to our study context, namely the Feasibility of Intervention Measure (FIM), Acceptability of Intervention Measure (AIM), and Intervention Appropriateness Measure (IAM) described in subsequent sections [64].

#### Aim 2: measures

The Feasibility of Intervention Measure (FIM), Acceptability of Intervention Measure (AIM), and Intervention Appropriateness Measure (IAM) are concise four-item assessments used to gauge implementation outcomes, serving as indicators of implementation success [64, 65]. These measures are instrumental in prospectively evaluating stakeholders’ perceptions regarding the feasibility, acceptability, and appropriateness of *Hoosier Sport Re-Social*. The FIM, AIM, and IAM have demonstrated robust psychometric properties, including content validity, discriminant content validity, reliability, structural validity, structural invariance, and responsiveness to change [64].

#### Aim 2: sample size calculation

To ensure a robust and effective co-design process, our teams will consist of faculty and student researchers (*n* = 6) collaborating with either adolescents (*n* = 5) or adults (parents, teachers) (*n* = 5) from the middle/high school community. Specifically targeting 6th–9th grade female students, generally aged 11 to 15 years. The composition of our teams reflects the unique expertise within our research group. The deliberate odd number of participants (totaling 13) serves a strategic purpose in facilitating decision-making processes during co-design sessions. This odd number ensures the possibility of a clear majority vote, which becomes instrumental in resolving potential ties when choosing between different design alternatives within the collaborative group. This thoughtful consideration of team composition and participant count underscores our commitment to fostering a productive and inclusive co-design environment for meaningful engagement and decision-making within the middle/high school community.

#### Aim 2: data analysis

The study will use inductive thematic analysis to analyze qualitative data from semi-structured interviews, aiming to gain insights directly from participants. This analysis involves identifying emerging patterns and themes without imposing predefined categories. Interviews will be transcribed verbatim, followed by an iterative coding process to identify meaningful units and group them into preliminary themes. Themes will be refined through team discussions to ensure accuracy and reliability. Inter-coder reliability will be established by multiple researchers coding a subset of interviews. The final thematic framework will offer a nuanced understanding of participants’ perspectives, informing recommendations crucial for the project’s outcomes. This analysis will serve as a robust basis for evidence-based conclusions and guide the project's direction.

Additionally, we will utilize descriptive statistics to analyze quantitative data derived from the Feasibility of Intervention Measure (FIM), Acceptability of Intervention Measure (AIM), and Intervention Appropriateness Measure (IAM) employed in session 5. To ensure a thorough assessment, we will establish clear criteria for evaluating the success of each feasibility aspect (a mean score of ≥ 16/20). Specifically, for the FIM, success will be determined by meeting a predefined threshold of participant engagement and adherence rates. For the AIM, we will gauge success based on whether participant satisfaction scores meet or exceed established benchmarks, indicating the intervention’s acceptability. The IAM will be evaluated by determining if the majority of participants consider the intervention appropriate based on predefined indicators of suitability and relevance. By the conclusion of session 5’s analysis, we will have a comprehensive draft of the intervention protocol prepared for piloting and feasibility testing in Aim 3. This draft will be informed by the results and success metrics from these measures, ensuring that the protocol is both feasible and well-received.

### Aim 3: To establish proof-of-concept and test Hoosier Sport Re-Social's feasibility, acceptability, and appropriateness

#### Aim 3: design

The third phase of the study will evaluate the feasibility of the Hoosier Sport Re-Social intervention (#NCT06556719) with 6th through 9th grade students from a rural middle or high school, conducted twice weekly over an 8-week period (ORBIT Phase Ib). Our assessment will focus on recommended feasibility measures for feasibility studies [28]. Alongside these measures, we will explore outcomes including self-reported weekly moderate-to-vigorous physical activity (MVPA) and physical literacy, body satisfaction, daily social media use duration, various psychosocial factors (such as self-esteem, body appreciation), and psychological needs using the BPNSFS.

Following the initial feasibility test, we will refine and retest the *Hoosier Sport Re-Social* intervention during the subsequent semester. To support intervention delivery, college student mentors—enrolled in upper division undergraduate or graduate public health programs—will collaborate with our research team. These mentors will undergo a 4-week training course before working with the middle or high school students. Their participation in delivering the intervention will be part of their academic coursework, earning them credit hours. The intervention is delivered by trained college students enrolled in upper-division public health and kinesiology courses. This model supports sustainability and scalability by leveraging an existing service-learning partnership between the university and the rural school. It also enhances relatability, as adolescents may feel more comfortable engaging with near-peer facilitators.

Our hypotheses for Aim 3 include several expectations: achieving full enrollment *(N* = 50), retaining 85% of participants by the intervention’s end, maintaining a 75% attendance rate, obtaining a mean score of ≥ 16 (considered a “good” score) on the Feasibility of Intervention Measure (FIM), AIM, and IAM, and ensuring an 80% fidelity rate in adhering to intervention procedures. Our selection of implementation measures (AIM, FIM, IAM) aligns with core constructs from the Theoretical Framework of Acceptability (TFA) and maps onto RE-AIM dimensions (e.g., implementation and acceptability). This approach supports a structured evaluation of the intervention’s feasibility and future scalability. The Hoosier Sport Re-Social intervention is described in accordance with the Template for Intervention Description and Replication (TIDieR) framework to enhance reproducibility. A structured fidelity checklist has also been developed to monitor delivery, adherence, and adaptations across implementation sessions, ensuring consistency with the intended intervention protocol. Progression to a full-scale randomized controlled trial (RCT) will be considered if the pilot study demonstrates the following: enrollment reaches at least 80% of the target sample (*N* = 50); retention at post-intervention assessment is 85% or higher; session attendance averages 75% or above; fidelity to the intervention protocol is maintained at or above 80% based on fidelity monitoring; and participants report mean scores of at least 16 out of 20 on validated measures of feasibility (FIM), acceptability (AIM), and appropriateness (IAM). These criteria align with recommendations for determining readiness to advance from a pilot to a full-scale trial in behavioral intervention research.

#### Aim 3: setting and sample

For the pilot study, we aim to recruit a total of *N* = 50 6th through 9th grade students. Similar to Aim 2, we will employ convenience sampling, striving for diversity in terms of biological sex and physical activity involvement. Our goal is to enroll an approximate 50% female and 50% male participant ratio, encompassing both regular and non-regular physical activity participants.

To be eligible for participation, students must meet specific criteria: currently enrolled in 6th through 9th grade at the school, possess parental consent to participate, provide their agreement to study participation (assent), commit to attending all school days during the intervention period, and be available for both baseline and post-intervention data collection. These criteria are crucial for the study’s integrity and aim to ensure comprehensive data collection and participation continuity.

#### Aim 3: procedure

The initial study recruitment details will be disseminated through email and newsletters by the research team. Adults interested in having their adolescent(s) participate will receive a Qualtrics survey link containing comprehensive study information. Eligibility confirmation will be communicated via email or phone. Upon receiving parental consent, a list of eligible adolescents will be generated for subsequent assent approaches. While risks are minimal, all physical activity sessions will be led by trained staff following school safety procedures. Any injuries or distress will be addressed immediately, with referrals made to school counselors if needed. Adverse events will be documented and reported to the IRB in accordance with institutional guidelines. All participant data will be de-identified and stored on password-protected, encrypted servers approved by the university. Only authorized research personnel will have access to identifiable information, which will be stored separately from survey or outcome data. Hard copy materials will be secured in locked cabinets. Identifiers will be removed from data prior to analysis and publication to ensure confidentiality.

A study information and recruitment session will be organized by the research team at the school site for adolescent participants. During this session, 6th–9th grade students will receive study information and an assent survey using Qualtrics. They will be encouraged to ask questions and informed of their right to discontinue participation at any time during the study. This session ensures that children are well-informed and comfortable with the study details before deciding to participate.

#### Aim 3: measures

**Fig. 5:**
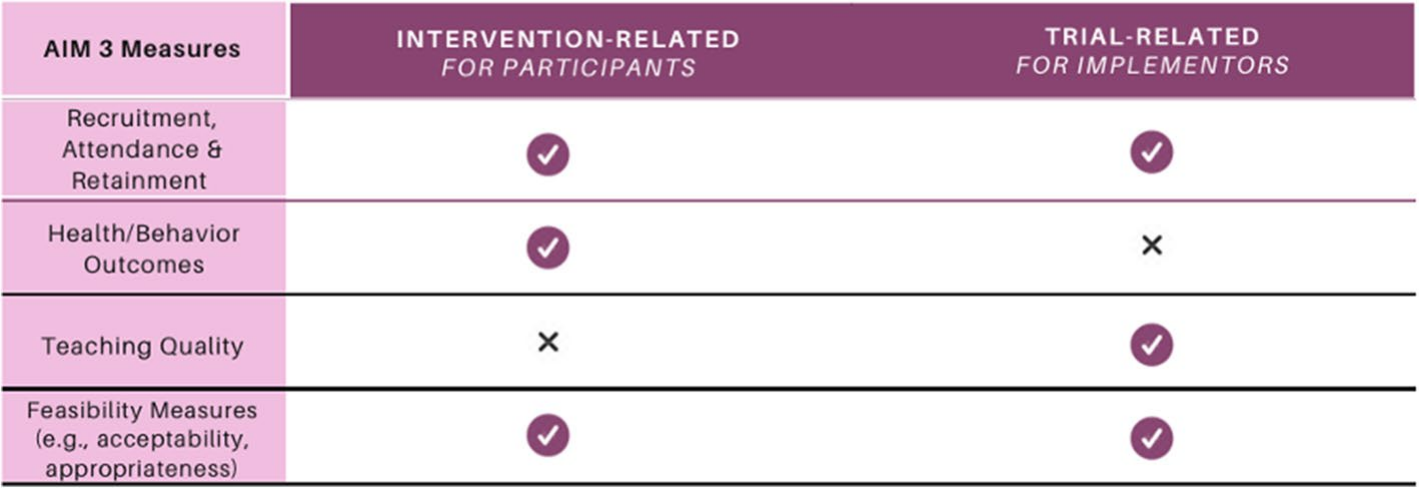
Intervention- and trial-related outcomes

Aligned with recently published feasibility research [28], we plan to evaluate two categories of feasibility measures: trial-related and intervention-related indicators. Our trial-related feasibility assessment will encompass gauging our capability for recruitment and retention. On the other hand, our intervention-related feasibility evaluation will involve measuring treatment fidelity, ensuring accurate, consistent, and high-quality delivery of intervention components. We will also assess the acceptability of the intervention, attendance rates, compliance (for instance, using weekly screen time report), cost considerations, and appropriateness, which involves evaluating setting, cultural norms, or specific requirements. These measures will be collected at two distinct intervals: mid-intervention and post-intervention—using Qualtrics recorded through mobile devices. This comprehensive evaluation framework will enable us to thoroughly assess both trial-related and intervention-related feasibility aspects of our study. Please see Fig. 5 for more details.

Additionally, preliminary behavior outcomes will be measured through replicating the measures of AIM 1 (BSQ, BAS-2, CAPL-2, pedometer wear, and a youth survey). These combined measures will provide data on potential changes from pre- to post-intervention in body satisfaction, body appreciation, physical literacy and activity levels, and social media usage.

##### Recruitment capability

Recruitment capability will be determined based on the number of children successfully enrolled into *Hoosier Sport Re-Social* (consent from parent/guardian and assent from child).

##### Retention

Retention will be measured based on the number of children who participate in the post-intervention data collection event. A make-up post-intervention event will be scheduled for child participants who miss the post-intervention data collection event.

##### Treatment fidelity

The evaluation of treatment fidelity in our study involves two primary stakeholders: the children participating in the intervention and the research team. Using self-report measures at both mid- and post-intervention points, we aim to understand how accurately, consistently, and effectively the intervention was delivered. This assessment process is guided by three key questions derived from prior research on school-based physical activity implementation [66]: first, *to what extent was the intervention delivered as planned?* Second, *how did the college student mentors adjust the program, if at all?* And third, *what were the underlying reasons for any adaptations made to the intervention?* By involving both the adolescents and the research team in this evaluation, we seek to gain comprehensive insights into the fidelity of the intervention delivery and any modifications made during its implementation.

##### Acceptability, appropriateness, and feasibility

Adolescents who participate in *Hoosier Sport Re-Social* will rate the feasibility, acceptability, and appropriateness of the intervention using the FIM, AIM, and IAM, respectively (each described in Aim 2).

##### Compliance

Screen time compliance will be assessed for each of the two physical activity data collections by determining the weekly average social media usage.

*Cost.* The cost of the intervention will be monitored throughout the study period and determined in comparison to the prospective study budget.

#### Aim 3: sample size calculation

A target sample size of 50 participants has been selected based on prior recruitment rates from the Hoosier Sport program, which has consistently engaged approximately 60 students per semester and achieved 100% retention over the past two semesters. This sample size will allow us to estimate feasibility indicators such as recruitment, retention, and intervention fidelity with reasonable precision. For example, a sample size of 50 would allow us to estimate a retention rate of 85% with a 95% confidence interval of ± 10%. This level of precision is appropriate for assessing progression criteria to a larger trial. Additionally, although not powered to detect statistical significance, this sample will support estimation of effect sizes related to body satisfaction, social media literacy, and psychosocial outcomes, which will inform future trial design and sample size calculations.

#### Aim 3: data analysis

##### Quantitative analysis of survey measures

In our analysis process, we will begin by reviewing the completeness and distributions of all variables. If any variables demonstrate non-normal distributions, we will apply normalizing transformations as required. Internal consistencies of scaled scores will be evaluated using Cronbach’s alpha to ensure reliability.

This study’s primary objective is to evaluate the intervention’s feasibility using the following indicators: Feasibility of Intervention Measure (FIM), Acceptability of Intervention Measure (AIM), Intervention Appropriateness Measure (IAM), attendance, and retention. Descriptive statistics—including means, standard deviations, and proportions—will be used to summarize these outcomes. A priori thresholds for success will be defined as a mean score of ≥ 16 out of 20 on the FIM, AIM, and IAM, representing a “good” rating. Attendance and retention rates will also be reported as proportions, with retention thresholds aligned with a minimum of 85% as an acceptable benchmark.

Secondary outcomes (e.g., body satisfaction and social media literacy) and exploratory outcomes (e.g., body appreciation and physical literacy) will be analyzed by assessing changes from pre- to post-intervention using paired-sample *t*-tests. Repeated measures ANOVAs will be used to further examine time effects and potential interactions. Age and BMI will be included as covariates in these models to enhance interpretation of between- and within-group differences.

#### Aim 3: missing data protocol

Given the exploratory nature of this pilot study, our primary approach to missing data will focus on descriptive assessment and transparent reporting rather than formal imputation. We will begin by identifying missing values and exploring patterns and extent of missingness across key variables. Where feasible, we will assess whether data are missing completely at random (MCAR), at random (MAR), or not at random (MNAR) to inform interpretation.

Consistent with current guidance for pilot studies, we will not use multiple imputation or other complex imputation techniques, as these require larger sample sizes to be valid and may introduce unnecessary complexity in a feasibility context. Instead, we will use complete case analysis for outcome analyses and report the number of participants included in each analysis. If substantial missingness is identified, we will describe it in detail and explore potential reasons, including participant characteristics or implementation factors, to inform future intervention refinement. All procedures will be transparently reported to support reproducibility and guide larger-scale trial planning.

## Discussion

Health-based interventions are more effective when they involve key community members in the planning process, as supported by various studies [67, 68]. Indeed, human-centered design is highlighted as crucial for addressing health challenges and promoting equitable healthcare solutions [69]. Previous research has shown that community members participating in co-design is effective for crafting innovative interventions, particularly in unique populations such as rural low-socioeconomic communities [62, 70]. Implementing this research design for an intervention aimed at enhancing body satisfaction among rural adolescent females has the potential to enhance the feasibility of the biopsychosocial intervention. Integrating community feedback into the intervention could foster an increased sense of acceptability within the community. Moreover, the unique feedback from the rural community may play a crucial role in addressing the gap in prior research on interventions addressing social media usage in this specific population.

Our human-centered protocol forms the basis for creating a feasible intervention in line with Phases Ia (define) and IIa (refine) of the ORBIT Model. This approach can be widely applied by researchers developing and piloting biopsychosocial interventions in school settings. Published evidence demonstrates that participatory co-design leads to effective intervention development [71, 72]. While co-design has been explored in physically active contexts involving adolescents, its application in the biopsychosocial field is still relatively new, and its application to body image and social media usage is novel [21, 22].

Our proposed methods account for the distinct needs, objectives, opportunities, and resources related to body image within the rural context, involving adolescents, parents, and teachers/administrators. This methodology aims to develop a physical activity-based intervention specifically customized for the middle and high school communities. In addressing both trial- and intervention-related feasibility indicators, our *Hoosier Sport Re-Social* protocol adheres to reporting feasibility measures in feasibility studies. This approach can contribute to future refinements in later phases of the ORBIT model.

## Supplementary Information

Supplementary Material 1: AIM 1 Body-Image Needs Assessment

## Data Availability

Not applicable (this manuscript does not report data generation or analysis).

## Abbreviations

BSQ: Body Shape Questionnaire
BAS-2: Body Appreciation Scale (version 2)
CAPL-2: Canadian Assessment of Physical Literacy (version 2)
GAD-7: Generalized Anxiety Disorder Assessment (7-item)
BSMAS: Bergen Social Media Addiction Scale
FIM: Feasibility of Intervention Measure
AIM: Acceptability of Intervention Measure
IAM: Intervention Appropriateness Measure

## Footnotes

1 “Hoosier” refers to individuals from the state of Indiana, reflecting the program’s geographic origin and community-based foundation. “Re-Social” refers to the program’s focus on helping youth re-engage in positive social, physical, and cognitive behaviors through sport, particularly in the context of rising digital media influence.
