# Supplementary material for: Hoosier Sport Re-Social: a protocol for developing a biopsychosocial body satisfaction intervention in rural Indiana
Source: Pilot Feasibility Stud. 2025 Aug 25;11:113. doi: 10.1186/s40814-025-01695-5 (PMC12376494; doi:10.1186/s40814-025-01695-5)
Supplement: Supplementary file 1 — Supplementary Material 1: AIM 1 Body-Image Needs Assessment [file 40814_2025_1695_MOESM1_ESM.pdf]

AIM 1 Body-Image Needs Assessment

Start of Block: Welcome

Q48 **Hoosier Sport Survey**
Hello and welcome to our exciting survey of students in the 6th through 9th grade! We are doing a research study. A research study is a special way to learn about something. We are doing this research study because we are trying to test a physical activity and mental health-related program at your school so we can help kids like you to develop healthy habits. We would like to ask you to be in this research study.

Why am I being asked to be in this research study?
You are being asked to be in this research study because you are a 6th-9th grade student. We value you and will use your thoughts and opinions to this program which could be successful in the future.

What will happen during this research study?
We want to tell you about some things that might happen if you are in the study. This study will take place at your school. We think it will last for 15 minutes. If you want to be in this study, you will complete a survey asking questions about:
how you use social media.
how you feel. how you view yourself.
what your physical activity levels are like.

Are there any bad things that might happen during the research study?
Sometimes bad things happen to people who are in research studies. These bad things are called "risks." The risks of being in this study might be that people could find out information you share in the survey. It is also possible that you could feel uncomfortable talking about some topics, and if this happens, you can choose to not answer any question you don't want to.

Are there any good things that might happen during the research study?
Sometimes good things happen to people who are in research studies. These good things are called "benefits." The benefits of being in this study might be:
Helping Others Feel Happier: Share your thoughts to make a positive impact on people's well-being.
Learning More About Yourself:
Understand your social media usage and physical activity levels better.
Being a Guide for Something New: Have a say in developing a new program at your school.

There is no cost to participate in the study.

To thank you for your participation in completing the survey, we will give you a $10 gift card.
How Will My Information be Used?
Some researchers and special groups might need to use your health info for their studies. They could share it with other researchers and even private companies. But don't worry, they will take out anything that shows who you are, like your name and contact details, before they share it. You don't need to give extra permission for this.

How Will My Information be Protected?
We will do our best to keep your personal information private, but we cannot promise complete confidentiality. We won't share any information that could be used to identify you in publications about this study. However, your personal information may be shared outside the research study as described above and/or if required by law.

Who can I ask if I have any questions?
If you have any questions about this study, you can ask the study coordinator, [PI name], at [PI phone number] or email them at [PI email]. You may also ask the research team the next time you see them.

What if I don't want to be in the study?
If you don't want to be in this study, you don't have to. It's up to you. If you say you want to be in it and then change your mind, that's OK. All you have to do is tell us that you don't want to be in it anymore. No one will be mad at you or upset with you if you don't want to be in it.

My choice: You will now be asked if you'd like to participate in the study. If you say yes, we will take that as verbal agreement to participate and that means you agree to participate in this research study. You do not need to sign anything to participate.

◯ I agree to participating in the study. (1)

◯ I do not agree to participating in the study. (2)

Skip To: End of Survey If Hoosier Sport Survey Hello and welcome to our exciting survey of students in the 6th through 9th... = I do not agree to participating in the study.

End of Block: Welcome

Start of Block: Phone Data

Q102 **SCREEN SHARING**
The next set of questions explore how you use your phone. You'll use the data stored on your phone to answer these questions as accurately as possible.

Screen time info **For these next questions, you'll have to go to your settings, and follow these steps:** *(1) go to your phone settings, (2) click on screen time, and (3) go to your daily average.*

phone avg **Please type in your "daily average" for total screen time (e.g., 9 in hours box, 14 in minutes box)**

○ Hours (4) ____

○ Minutes (5) ____

phone app. **Please type in your "most used" app name (e.g., safari)?**

____

Q182 **What three categories appear under the daily average? (e.g., Entertainment, Games, Social Networking)**

____

Q183 **Drag the sliders below to share your average pickups and notifications**

How many "pickups" do you have on average in a day? ()	
How many "notifications" do you have on average in a day? ()	

Q187 **What app is your "first use after pickup" most often?**

____

Q189 **What app sends you the most notifications?**

____

phone total **Under "screen time", Please report your average hours for each app**. *Type in the box on the right. Report in the format of: hours.minutes (e.g., 4 hours and 5 minutes = 4.5)*
Weekly average of Instagram use : _______ (1)
Weekly average of TikTok use : _______ (2)
Weekly average of YouTube use : _______ (3)
Weekly average of Snapchat use : _______ (4)
Weekly average of Be Real use : _______ (5)
Total : ________

End of Block: Phone Data

Start of Block: GAD7

Q171 **MENTAL HEALTH CHECK-IN**
The next few questions ask about your mental health. There are no right or wrong answers.

Q170 **Over the last two weeks**, how often have you been bothered by the following problems?

	Not at all (1)	Several days (2)	More than half the days (3)	Nearly every day (4)
Feeling nervous, anxious, or on edge (1)	○	○	○	○
Not being able to stop or control worrying (2)	○	○	○	○
Worrying too much about different things (3)	○	○	○	○
Trouble relaxing (4)	○	○	○	○
Being so restless that it is hard to sit still (5)	○	○	○	○
Becoming easily annoyed or irritable (6)	○	○	○	○
Feeling afraid, as if something awful might happen (7)	○	○	○	○

End of Block: GAD7

Start of Block: Body Perception and Social Media

Q106 **EXPLORING SOCIAL MEDIA EXPERIENCES**
Social media can make us feel good or bad. The next section of questions ask about social media or interactions with others on social media.

Q53 **Have you ever edited or filtered your photos before posting them on social media to change your appearance?**

- ○ No (7)
- ○ Yes (8)

Display This Question:
If Have you ever edited or filtered your photos before posting them on social media to change your a... = Yes

Q54 **How have you edited photos?** *Select all that apply.*

- ▢ Using a filter to change your facial features (nose, lips, eyes) (5)
- ▢ Using a filter to change your general appearance (hair color, skin color) (6)
- ▢ Using a filter or tool to change your body appearance (hip width, body length) (7)
- ▢ Other (8) ____

Q19 **Have you ever felt pressured to look a certain way because of images you've seen on social media?**

- ○ Yes (4)
- ○ No (5)

Display This Question:

If Have you ever felt pressured to look a certain way because of images you've seen on social media? = Yes

Q55 **Briefly describe a time you felt pressured to look a certain way because of an image you saw on social media (like feeling the need to have smaller or bigger body features because of someone you saw)**

____

Q17 **Has anyone ever said anything bad about your body on social media?**

- ◯ Yes (4)
- ◯ No (5)

Display This Question:

If Has anyone ever said anything bad about your body on social media? = Yes

Q18 **How did it make you feel?**

- ◯ Insecure about myself (4)
- ◯ Better about myself (5)
- ◯ It didn't affect me (6)

End of Block: Body Perception and Social Media

Start of Block: Bergen SM Scale

Q188 **Please indicate below how much you agree with each statement about social media.**

	Very Rarely (1)	Rarely (2)	Sometimes (3)	Often (4)	Very Often (5)
You spend a lot of time thinking about social media or planning how to use it. (1)	○	○	○	○	○
You feel an urge to use social media more and more (2)	○	○	○	○	○
You use social media in order to forget about personal problems (3)	○	○	○	○	○
You have tried to cut down on the use of social media without success (4)	○	○	○	○	○
You become restless or troubled if you are prohibited from using social media (5)	○	○	○	○	○
You use social media so much that it has had a negative impact on your job/studies (6)	○	○	○	○	○

End of Block: Bergen SM Scale

Start of Block: MAIA-Y

Q110 **<u>LISTENING TO OUR BODIES</u>**
The next set of questions ask you about how you feel/experience your body. There are no wrong answers!

Noticing **For the next few questions, choose the answer that best fits how often you do what the sentence says. Please choose ONE answer for EACH row.** Choose a number between 0 (never) and 5 (always) to tell us how often you do what the sentence says.

	Never (28)	Rarely (29)	Sometimes (30)	Often (31)	Very Frequently (32)	Almost Always (33)
When I am nervous, I can tell where in my body the feelings come from. (5)	○	○	○	○	○	○
I can tell when I am uncomfortable in my body. (6)	○	○	○	○	○	○
I can tell where in my body I am comfortable. (7)	○	○	○	○	○	○
I can tell when my breathing changes, like if it slows down or speeds up. (19)	○	○	○	○	○	○

Not Distracting **For the next few questions, choose the answer that best fits how often you do what the sentence says. Please choose ONE answer for EACH row.** Choose a number between 0 (never) and 5 (always) to tell us how often you do what the sentence says.

	Never (28)	Rarely (29)	Sometimes (30)	Often (31)	Very Frequently (32)	Almost Always (33)
I ignore bad feelings in my body until they become very strong. (5)	○	○	○	○	○	○
I distract myself when I feel uncomfortable or feel pain. (6)	○	○	○	○	○	○
When I feel uncomfortable or feel pain, I try to get over it. (7)	○	○	○	○	○	○

Not Worrying **Please choose ONE answer for EACH row.** *Choose a number between 0 (never) and 5 (always) to tell us how often you do what the sentence says.*

	Never (17)	Rarely (18)	Sometimes (19)	Often (20)	Very Frequently (21)	Almost Always (22)
When I feel pain in my body, I become upset (1)	○	○	○	○	○	○
I get worried if I feel pain or if I feel uncomfortable (2)	○	○	○	○	○	○
I can tell if I have a bad feeling in my body but I don't worry about it (3)	○	○	○	○	○	○

Attention Reg. **Please choose ONE answer for EACH row.** *Choose a number between 0 (never) and 5 (always) to tell us how often you do what the sentence says.*

	Never (9)	Rarely (10)	Sometimes (11)	Often (12)	Very Frequently (13)	Almost Always (14)
I can focus on how I breathe without thinking about anything else (1)	○	○	○	○	○	○
I can focus on the feelings in my body, even when there is a lot going on around me (2)	○	○	○	○	○	○
When I am talking to someone, I can focus on the way I am standing or sitting (10)	○	○	○	○	○	○
Even if I am distracted I can go back to thinking how my body feels (3)	○	○	○	○	○	○
I can return my focus from thinking about things to feeling my body (4)	○	○	○	○	○	○

I can pay attention to my whole body even when a part of it is in pain (5)	○	○	○	○	○	○
I can focus on my entire body when I try (6)	○	○	○	○	○	○

Emotional Aware. **Please choose ONE answer for EACH row.** *Choose a number between 0 (never) and 5 (always) to tell us how often you do what the sentence says.*

	Never (9)	Rarely (10)	Sometimes (11)	Often (14)	Very Frequently (12)	Almost Always (13)
I can feel how my body changes when I am angry (1)	○	○	○	○	○	○
When something is wrong in my life I can feel it in my body (2)	○	○	○	○	○	○
After a peaceful moment, I can feel my body is different (10)	○	○	○	○	○	○
I can feel that my breathing becomes free and easy when I am comfortable (3)	○	○	○	○	○	○
I can feel how my body changes when I feel happy (4)	○	○	○	○	○	○

Self-Reg. **Please choose ONE answer for EACH row.** *Choose a number between 0 (never) and 5 (always) to tell us how often you do what the sentence says.*

	Never (9)	Rarely (10)	Sometimes (11)	Often (14)	Very Frequently (12)	Almost Always (13)
I can feel calm even if there is a lot going on (1)	○	○	○	○	○	○
When I focus on how I feel in my body, I calm down. (2)	○	○	○	○	○	○
I can use my breath to help me calm down and relax. (10)	○	○	○	○	○	○
When I am thinking too much, I can calm my mind by focusing on my body/breathing (3)	○	○	○	○	○	○

Body Listening **Please choose ONE answer for EACH row.** *Choose a number between 0 (never) and 5 (always) to tell us how often you do what the sentence says.*

	Never (9)	Rarely (10)	Sometimes (11)	Often (14)	Very Frequently (12)	Always (13)
I listen for clues from my body about my emotions (1)	○	○	○	○	○	○
When I focus on how I feel in my body, I calm down (2)	○	○	○	○	○	○
I can use my breath to help me calm down and relax (10)	○	○	○	○	○	○
When I am thinking too much, I can calm my mind by focusing on my body/breathing (3)	○	○	○	○	○	○

Trusting **Please choose ONE answer for EACH row.** *Choose a number between 0 (never) and 5 (always) to tell us how often you do what the sentence says.*

	Never (9)	Rarely (10)	Sometimes (11)	Often (14)	Very Frequently (12)	Almost Always (13)
I feel good in my body (1)	○	○	○	○	○	○
I feel my body is a safe place (2)	○	○	○	○	○	○
I trust the way my body feels (10)	○	○	○	○	○	○

Q111 **APPRECIATING OURSELVES**
The next set of questions asks about how you appreciate your body. There are no wrong answers!

BAS1 How often are each of the following statements true for you? *Please choose ONE answer for EACH row.*

	Never (1)	Seldom (2)	Sometimes (3)	Often (4)	Always (5)
I respect my body (BAS_1)	○	○	○	○	○
I feel good about my body (BAS_2)	○	○	○	○	○
On the whole, I am satisfied with my body. (BAS_3)	○	○	○	○	○
Despite its flaws, I accept my body for what it is (BAS_4)	○	○	○	○	○
I feel that my body has at least some good qualities (BAS_5)	○	○	○	○	○
I take a positive attitude towards my body (BAS_6)	○	○	○	○	○
I am attentive to my body's needs (BAS_7)	○	○	○	○	○
My self worth is independent of my body shape and weight (BAS_8)	○	○	○	○	○

I do not focus a lot of energy being concerned with my body's shape or weight (BAS_9)	○	○	○	○	○
My feelings toward my body are positive for the most part (BAS_10)	○	○	○	○	○
I engage in healthy behaviors to take care of my body (BAS_11)	○	○	○	○	○
I do not allow unrealistically thin images of women/men presented in the media to affect my attitudes toward my body (BAS_12)	○	○	○	○	○
Despite its imperfections I still like my body (BAS_13)	○	○	○	○	○

End of Block: Body Appreciation Scale 2 (BAS-2)

Start of Block: Body Shape Questionnaire (BSQ)

Q113 **BODY SHAPE AND PERSPECTIVE.**
The next set of questions explore the diverse perspectives on body shape. These questions aim to gather insights into how people perceive and relate to their own bodies. There are no wrong answers!

Q114 We would like to know how you have been feeling about your appearance over the PAST FOUR WEEKS. *Please read each question and choose one answer for each row.*

	Never (1)	Rarely (2)	Sometimes (3)	Often (4)	Very Often (5)	Always (6)
Has feeling bored made you over think about your shape? (Q114 _1)	○	○	○	○	○	○
Have you been so worried about your shape that you have been feeling you ought to diet? (Q114 _2)	○	○	○	○	○	○
Have you thought that your thighs, hips or bottom are too large for the rest of you? (Q114 _3)	○	○	○	○	○	○
Have you been afraid that you might become fat (or fatter)? (Q114 _4)	○	○	○	○	○	○
Have you worried about your flesh being not firm enough? (Q114 _5)	○	○	○	○	○	○
Has feeling full (e.g. after eating a large meal) made you feel fat? (Q114 _6)	○	○	○	○	○	○

Q115 We would like to know how you have been feeling about your appearance over the PAST FOUR WEEKS. *Please read each question and choose one answer for each row.*

	Never (1)	Rarely (2)	Sometimes (3)	Often (4)	Very Often (5)	Always (6)
Have you felt so bad about your shape that you have cried? (Q115_1)	o	o	o	o	o	o
Have you avoided running because your flesh might wobble? (Q115_2)	o	o	o	o	o	o
Has being with thin people made you feel self-conscious about your shape? (Q115_3)	o	o	o	o	o	o
Have you worried about your thighs spreading out when sitting down? (Q115_4)	o	o	o	o	o	o
Has eating even a small amount of food made you feel fat? (Q115_5)	o	o	o	o	o	o
Have you noticed the shape of other people and felt that your own shape compared unfavourably? (Q115_6)	o	o	o	o	o	o

Q116 We would like to know how you have been feeling about your appearance over the PAST FOUR WEEKS. *Please read each question and choose one answer for each row.*

	Never (1)	Rarely (2)	Sometimes (3)	Often (4)	Very Often (5)	Always (6)
Has thinking about your shape interfered with your ability to concentrate (e.g. while watching television, reading, listening to conversations)? (1)	○	○	○	○	○	○
Have you avoided wearing clothes which make you particularly aware of the shape of your body? (3)	○	○	○	○	○	○
Have you worried about your thighs spreading out when sitting down? (4)	○	○	○	○	○	○
Has eating sweets, cakes, or other high calorie food made you feel fat? (8)	○	○	○	○	○	○

Q117 We would like to know how you have been feeling about your appearance over the PAST FOUR WEEKS. *Please read each question and choose one answer for each row.*

	Never (1)	Rarely (2)	Sometimes (3)	Often (4)	Very Often (5)	Always (6)
Have you not gone out to social occasions (e.g. parties) because you have felt bad about your shape? (1)	○	○	○	○	○	○
Have you felt excessively large and rounded? (2)	○	○	○	○	○	○
Have you felt ashamed of your body? (3)	○	○	○	○	○	○
Has worry about your shape made you diet? (4)	○	○	○	○	○	○
Have you felt happiest about your shape when your stomach has been empty (e.g. in the morning)? (5)	○	○	○	○	○	○

Q118 We would like to know how you have been feeling about your appearance over the PAST FOUR WEEKS. *Please read each question and choose one answer for each row.*

	Never (1)	Rarely (2)	Sometimes (3)	Often (4)	Very Often (5)	Always (6)
Have you worried about other people seeing rolls of fat around your waist or stomach? (1)	○	○	○	○	○	○
Have you felt that it is not fair that other people are thinner than you? (2)	○	○	○	○	○	○
Have you vomited in order to feel thinner? (3)	○	○	○	○	○	○
When in company have your worried about taking up too much room (e.g. sitting on a sofa, or a bus seat)? (4)	○	○	○	○	○	○
Have you worried about your skin being dimply? (5)	○	○	○	○	○	○

Q119 We would like to know how you have been feeling about your appearance over the PAST FOUR WEEKS. *Please read each question and choose one answer for each row.*

	Never (1)	Rarely (2)	Sometimes (3)	Often (4)	Very Often (5)	Always (6)
Have you pinched areas of your body to see how much fat there is? (1)	o	o	o	o	o	o
Have you avoided situations where people could see your body (e.g. communal changing rooms or swimming baths)? (2)	o	o	o	o	o	o
Have you been particularly self-conscious about your shape when in the company of other people? (4)	o	o	o	o	o	o
Has worry about your shape made you feel you ought to exercise? (5)	o	o	o	o	o	o

Q172 **PHYSICAL ACTIVITY**
The next few questions ask about your physical activity levels.

Q173 Thinking about vigorous physical activities you did in the last 7 days, did you do vigorous activities for at least 10 minutes at a time, such as running, aerobics, sports, heavy yard work, or anything else?

	0	1	2	3	4	5	6	7
Drag the bar to select your answer in days ()								

Q177 On the days when you do vigorous activity for at least 10 minutes, how long do you do those activities?

- ○ About 10 minutes (1)
- ○ About 20 minutes (2)
- ○ About 30 minutes (3)
- ○ About 40 minutes (4)
- ○ About 50 minutes (5)
- ○ 1 hour or more (6)

Q174 Thinking about moderate physical activities you did in the last 7 days, did you do moderate activities for at least 10 minutes at a time, such as brisk walking, bicycling at regular pace, vacuuming, gardening, or anything else?

	0	1	2	3	4	5	6	7
Drag the bar to select your answer in days ()								

Q179 On the days when you do moderate activity for at least 10 minutes, how long do you do those activities?

- About 10 minutes (1)
- About 20 minutes (2)
- About 30 minutes (3)
- About 40 minutes (4)
- About 50 minutes (5)
- 1 hour or more (6)

Q175 Now thinking about the amount of time you spent walking in the last 7 days, that lasted for at least 10 minutes at a time. This includes at work and at home, walking to travel from place to place, and any other walking that you might do solely for recreation, sport, exercise, or leisure

	0	1	2	3	4	5	6	7
Drag the bar to select your answer in days ()								

On the days when you do walk for at least 10 minutes, how long do you do those activities?

- About 10 minutes (1)
- About 20 minutes (2)
- About 30 minutes (3)
- About 40 minutes (4)
- About 50 minutes (5)
- 1 hour or more (6)

Q176 Now thinking about the amount of time you spent sitting in the last 7 days. Include time spent at work, at home, while doing course work and during leisure time. This may include time spent sitting at a desk, visiting friends, reading, or sitting or lying down to watch television

- ◯ About 30 minutes or less (1)
- ◯ About 1 hour (2)
- ◯ About 2 hours (3)
- ◯ About 3 hours (4)
- ◯ About 4 Hours (5)
- ◯ About 5 hours or more (6)

Q146 **How do you exercise most often?**

- ◯ On a sports team (1)
- ◯ Doing exercises at a gym or at home (2)
- ◯ Playing sports with people I know but not on an organized team (4)
- ◯ walking, biking, or skate boarding in my neighborhood or other places (5)
- ◯ Gym class at school (6)

Start of Block: CAPL-2 Coded Questions

JS

Q1 Here is a sample question for you to try.

First, choose the sentence that is more like you.

THERE ARE NO RIGHT OR WRONG ANSERS, JUST TELL US WHAT YOU THINK IS **MOST LIKE YOU!**

- ◯ Some kids like to play with computers (1)
- ◯ Other kids don't like playing with computers (2)

Display This Question:

If Here is a sample question for you to try. First, choose the sentence that is more like you. THERE... = Some kids like to play with computers

Or Here is a sample question for you to try. First, choose the sentence that is more like you. THERE... = Other kids don't like playing with computers

Q2 Is "${Q1/ChoiceGroup/SelectedChoices}" REALLY TRUE for you or SORT OF TRUE for you?

◯ REALLY TRUE for me (1)

◯ SORT OF TRUE for me (2)

Q3 Now, we're going to ask you to read a story about Sally. This story about Sally is missing some words. Each screen will ask you to fill in a different word in the story. You will select a word from the box shown below to fill in the missing word that is shown as (1), (2), (3), etc. in the story. Each word can only be used once to fill one blank space in the story. There are more words than blank spaces so not all words will be used.

Q4 Sally tries to be active every day. Running every day is good for her heart and her lungs. *Sally thinks that physical activity is* ***(1)*** *and is also* ***(2)*** *for her.* At her sport team's practice she does more running to improve her **(3)**. The team also does exercises like push-ups and sit-ups that increase her **(4)**. When cooling down, she **(5)** to improve her flexibility and slow her heart rate. After exercising, she checks her heart rate which is also called a **(6)**.

Q5 Fill in **(1)** above:

▼ fun (18) ... sport (38)

Q6 Sally tries to be active every day. Running every day is good for her heart and her lungs. *Sally thinks that physical activity is **${Q5/ChoiceGroup/SelectedChoices}** and is also **(2)** for her.* At her sport team's practice she does more running to improve her **(3)**. The team also does exercises like push-ups and sit-ups that increase her **(4)**. When cooling down, she **(5)** to improve her flexibility and slow her heart rate. After exercising, she checks her heart rate which is also called a **(6)**.

Carry Forward Unselected Choices from "Fill in (1) above:"

Q7 Fill in **(2)** above:

▼ fun (1) ... sport (10)

Q8 Sally tries to be active every day. Running every day is good for her heart and her lungs. Sally thinks that physical activity is **${Q5/ChoiceGroup/SelectedChoices}** and is also **${Q7/ChoiceGroup/SelectedChoices}** for her. *At her sport team's practice she does more running to improve her **(3)**.* The team also does exercises like push-ups and sit-ups that increase her **(4)**. When cooling down, she **(5)** to improve her flexibility and slow her heart rate. After exercising, she checks her heart rate which is also called a **(6)**.

Carry Forward Unselected Choices from "Fill in (2) above:"

Q9 Fill in **(3)** above:

▼ fun (1) ... sport (10)

Q10 Sally tries to be active every day. Running every day is good for her heart and her lungs. Sally thinks that physical activity is **${Q5/ChoiceGroup/SelectedChoices}** and is also **${Q7/ChoiceGroup/SelectedChoices}** for her. At her sport team's practice she does more running to improve her **${Q9/ChoiceGroup/SelectedChoices}**. *The team also does exercises like push-ups and sit-ups that increase her **(4)**.* When cooling down, she **(5)** to improve her flexibility and slow her heart rate. After exercising, she checks her heart rate which is also called a **(6)**.

Carry Forward Unselected Choices from "Fill in (3) above:"

Q11 Fill in **(4)** above:

▼ fun (1) ... sport (10)

Q12 Sally tries to be active every day. Running every day is good for her heart and her lungs. Sally thinks that physical activity is **${Q5/ChoiceGroup/SelectedChoices}** and is also **${Q7/ChoiceGroup/SelectedChoices}** for her. At her sport team's practice she does more running to improve her **${Q9/ChoiceGroup/SelectedChoices}**. The team also does exercises like push-ups and sit-ups that increase her **${Q11/ChoiceGroup/SelectedChoices}**. *When cooling down, she **(5)** to improve her flexibility and slow her heart rate.* After exercising, she checks her heart rate which is also called a **(6)**.

Carry Forward Unselected Choices from "Fill in (4) above:"

Q13 Fill in **(5)** above:

▼ fun (1) ... sport (10)

Q14 Sally tries to be active every day. Running every day is good for her heart and her lungs. Sally thinks that physical activity is **${Q5/ChoiceGroup/SelectedChoices}** and is also **${Q7/ChoiceGroup/SelectedChoices}** for her. At her sport team's practice she does more running to improve her **${Q9/ChoiceGroup/SelectedChoices}**. The team also does exercises like push-ups and sit-ups that increase her **${Q11/ChoiceGroup/SelectedChoices}**. When cooling down, she **${Q13/ChoiceGroup/SelectedChoices}** to improve her flexibility and slow her heart rate. *After exercising, she checks her heart rate which is also called a **(6)**.*

Carry Forward Unselected Choices from "Fill in (5) above:"

Q15 Fill in **(6)** above:

▼ fun (1) ... sport (10)

Q16 Here is the final paragraph with all your choices:

Sally tries to be active every day. Running every day is good for her heart and her lungs. Sally thinks that physical activity is **${Q5/ChoiceGroup/SelectedChoices}** and is also **${Q7/ChoiceGroup/SelectedChoices}** for her. At her sport team's practice she does more running to improve her **${Q9/ChoiceGroup/SelectedChoices}**. The team also does exercises like push-ups and sit-ups that increase her **${Q11/ChoiceGroup/SelectedChoices}**. When cooling down,
she **${Q13/ChoiceGroup/SelectedChoices}** to improve her flexibility and slow her heart rate. After exercising, she checks her heart rate which is also called a **${Q15/ChoiceGroup/SelectedChoices}**.

Hit next to move to the next part of the survey.

End of Block: CAPL-2 Coded Questions

Start of Block: Demographics

Q108 **TELL US ABOUT YOU!**
You're almost done with this survey! The last questions focus on basic information about you (e.g., age, grade).

Age **How old are you?**

- ◯ 10 years old (1)
- ◯ 11 years old (2)
- ◯ 12 years old (3)
- ◯ 13 years old (4)
- ◯ 14 years or older (5)

Grade **What grade are you in?**

- ◯ 6th grade (1)
- ◯ 7th grade (2)
- ◯ 8th grade (3)
- ◯ 9th grade (4)

Sex **Are you a:**

○ Male (1)

○ Female (2)

○ I use a different term (4) ____

Ethnicity **Please select the option that best describes your ethnicity:**

○ White (1)

○ Black (2)

○ Hispanic or Latino (3)

○ Native American or Alaska Native (4)

○ Native Hawaiian or Pacific Islander (5)

○ Other (please specify) (6) ____

End of Block: Demographics

Q138 **You're all done! Thanks for contributing to science!**

End of Block: Ending
